# Methicillin-resistant *Staphylococcus aureus* nasal colonization among HIV-infected patients in Taiwan: prevalence, molecular characteristics and associated factors with nasal carriage

**DOI:** 10.1186/s12879-020-04979-8

**Published:** 2020-03-30

**Authors:** Yi-Yu Hsu, David Wu, Chien-Ching Hung, Shie-Shian Huang, Fang-Hsueh Yuan, Ming-Hsun Lee, Ching-Tai Huang, Shian-Sen Shie, Po-Yen Huang, Chien-Chang Yang, Chun-Wen Cheng, Hsieh-Shong Leu, Ting-Shu Wu, Yhu-Chering Huang

**Affiliations:** 1 Department of Ophthalmology, Mackay Memorial Hospital, Taipei, Taiwan; 2 Department of Internal Medicine, Cathay General hospital, Taipei, Taiwan; 3 Department of Internal Medicine, National Taiwan University Hospital and National Taiwan University College of Medicine, Taipei, Taiwan; 4 Department of Medicine, Chang Gung University School of Medicine, Kweishan, Taoyuan, Taiwan; 5 Division of Infectious Diseases, Chang Gung Memorial Hospital at Keelung, Keelung, Taiwan; 6 Division of Infectious Diseases, Chang Gung Memorial Hospital at Linkou, Kweishan, Taoyuan, Taiwan; 7 Division of Pediatric Infectious Diseases, Chang Gung Memorial Hospital at Linkou, Kweishan, Taoyuan, Taiwan; 8 Department of Pediatrics, Chang Gung Memorial Hospital, No. 5, Fu-Shin Street, Kweishan, 333 Taoyuan, Taiwan

**Keywords:** Nasal carriage, Antibiotic use, Injection drug use, Injection drug user, Molecular typing

## Abstract

**Background:**

To evaluate nasal carriage, antibiotic susceptibility and molecular characteristics of methicillin-resistant *Staphylococcus aureus* (MRSA), as well as the risk factors of MRSA colonization, in human immunodeficiency virus (HIV)-infected patients in northern Taiwan.

**Methods:**

From September 2014 to November 2015, HIV-infected patients seeking outpatient care at four hospitals were eligible for this study. A nasal specimen was obtained from each subject for the detection of *S. aureus* and a questionnaire was completed by each subject. MRSA isolates once identified were characterized.

**Results:**

Of 553 patients surveyed, methicillin-susceptible *S. aureus* (MSSA) was detected in 119 subjects (21.5%) and MRSA in 19 subjects (3.4%). Female gender, injection drug use, smoking, hepatitis C virus carrier, cancer and antibiotic use within 1 year were positively associated with MRSA colonization. By multivariate analysis, only cancer (adjust odds ratio (aOR) 7.78, [95% confidence interval (CI), 1.909–31.731]) and antibiotic use within 1 year (aOR 3.89, [95% CI, 1.219–12.433]) were significantly associated with MRSA colonization. Ten isolates were characterized as sequence type (ST) 59/staphylococcal chromosome cassette (SCC) IV or V_T_, endemic community strains in Taiwan, four isolates as ST 8/SCC*mec* IV (USA 300) and one isolate as ST 239/SCC*mec* IIIA, a hospital strain. All the community-associated MRSA isolates were susceptible to trimethoprim-sulfamethoxazole (TMP-SMX).

**Conclusions:**

Nasal MRSA carriage in HIV-infected patients seeking outpatient care was low (3.4%) in northern Taiwan. Most of the colonizing isolates were genetically endemic community strains and exhibited high susceptibility to TMP-SMX and fluoroquinolones. Cancer and antibiotic use within 1 year were associated with MRSA colonization.

## Background

Methicillin-resistant *Staphylococcus aureus* (MRSA) was first reported in the 1960s [1] and the infections due to MRSA rapidly increased in the 1980s [2–4], most of which were health care-acquired infections. In the late 1990s, MRSA began to be recognized as a major cause of community-onset infections, which was termed as community-associated MRSA (CA-MRSA) later [2–4]. The epidemic of CA-MRSA has spread rapidly both in the community [2–4] and in healthcare settings over the past decade. Carriage of *S. aureus*, including MRSA, has been identified as a predisposing factor for subsequent invasive infections [5, 6], and the anterior nares are the most common site of colonization.

Among human immunodeficiency virus (HIV)-infected patients, *S. aureus* infection is considered to be a major cause of significant morbidity and mortality [7–9]. Previous studies suggest that prevalence of nasal colonization with MRSA is higher in HIV-infected individuals than in the general population [10–12]. HIV infection is also related to persistent colonization [13]. The higher colonization burden may be associated with a higher incidence of subsequent infections [10, 11, 14]. However, risk factors for *S. aureus* colonization appear to be different among different populations, including patients infected with HIV [11, 15–17]. MRSA colonization in HIV-infected patients may vary widely in different geographical regions [15, 18–21], the time point of survey [14], and the coverage of antiretroviral therapy in the population [22].

MRSA has been a prevalent etiology of infections in Asian countries, including Taiwan, either in healthcare settings or in the community, for decades, but the reports regarding MRSA colonization in HIV-infected population have been scarce [12, 15, 23]. Hence, we conducted this study to evaluate the prevalence of and associated factors for MRSA colonization in HIV-infected population in Taiwan. All collected MRSA isolates were further characterized by molecular methods and antibiotic susceptibility.

## Methods

The study was approved by the institutional review board (IRB) of Chang Gung Memorial Hospital (protocol number:103-2418A3) and the Research Ethics Committee of National Taiwan University Hospital. A written informed consent was obtained from each subject.

### Subject enrollment

The study was conducted in hospital-based infectious diseases outpatient clinics at Chang Gung Memorial Hospital (CGMH) and National Taiwan University Hospital (NTUH) from September 2014 to November 2015. Both CGMH and NTUH are university-affiliated medical centers situated in northern Taiwan. Data were obtained in multiple CGMH branches, including Linkou branch, Taipei branch and Keelung branch. We also recruited patients from prison and correctional facilities in Keelung, where inmates were regularly followed by infectious diseases specialists in CGMH Keelung branch. All the HIV-infected patients who visited and treated at CGMH and NTUH were eligible and invited to participate in this study. Study subjects were enrolled when they sought HIV care at the infectious diseases outpatient departments.

### Data collection

To identify the associated factors with MRSA acquisition, a self-administered questionnaire interview was performed to inquire into the factors for colonization with MRSA in HIV-infected patients. The following information was also collected from medical records of the participants: age, gender, drinking habits and smoking habits, underlying diseases, clinical characteristics, latest hospitalization, community exposure (eg, drug use, and incarceration), previous *S. aureus* infection, antibiotic use, skin disease, plasma HIV RNA load and CD4 + T lymphocytes, and antiretroviral therapy. Clinical information regarding hospitalization, residence in a long-term care facility, outpatient department visit, surgery, dialysis, and usage of tubes (nasogastric tube, urine catheter, tracheostomy tube, drainage tube, port-A, and dialysis tube) were also obtained. Substance abuse was defined as current or former use if illicit drugs.

### Microbiologic study

Nasal specimens were obtained by swabbing the anterior 1 cm of the nasal vestibule of both anterior nares of the participants. The swabs were preserved in the transport medium (Venturi Transystem, Copan Innovation Ltd.) immediately. *S. aureus* was identified by colony morphology, Gram stain, positive coagulase tests, and presence of β-hemolysis after subculture to tryptic soy agar plates containing 5% sheep’s blood. Methicillin resistance was confirmed by cefoxitin disk-diffusion method according to the recommendation of Clinical and Laboratory Standard Institutes [24].

### Antimicrobial susceptibility study

The antimicrobial susceptibility of all MRSA isolates to ten antibiotics, including ciprofloxacin, trimethoprim/sulfamethoxazole (SXT), penicillin, teicoplanin, linezolid, clindamycin, doxycyclin, fusidic acid, vancomycin, and erythromycin, was tested in accordance with the guideline of Clinical and Laboratory Standard Institutes by using the disk-diffusion method [24].

### Molecular characterization

All the MRSA isolates, once identified, were characterized by molecular methods. By pulsed-field gel electrophoresis (PFGE) with *Sma*I digestion [25–27], the genotypes were designated in alphabetical order, as in our previous studies [25–27]; any new genotype, if identified, was designated consecutively. PFGE patterns with fewer than four band differences from an existing genotype were defined as subtypes of that genotype. Staphylococcal chromosome cassette mec (SCC*mec*) type, and the presence of Panton-Valentine leukocidin (PVL) genes were determined by a multiplex PCR strategy [28]. Some isolates of representative PFGE patterns were selected and underwent multilocus sequence typing (MLST) [29] and *spa* typing [27]. The allelic profiles were assigned through comparison of the sequences at each locus with those of the known alleles in the *S. aureus* MLST database and were defined as sequence types accordingly. The details of the procedures were described elsewhere previously [25–29].

### Statistical analysis

Statistical analyses were performed with the Statistical Package for the Social Sciences (SPSS software for Windows, version 17.0). The definition of statistical significance was *p* < 0.05. Chi-square analysis or Fisher’s exact test was used for examination of categorical variables. Continuous variables were compared between patients with MRSA colonization versus patients without MRSA colonization using two-sample t-test, in which Levene’s test was used to determine equality of variances between the two groups.

## Results

During the 14-month study period, a total of 810 HIV-infected patients sought outpatient care at CGMH and NTUH (446 from CGMH and 364 from NTUH). Of the 810 eligible patients, 586 patients (275 from CGMH and 311 from NTUH) were interviewed and invited to participate in this study. Five hundred fifty-three patients (259 from CGMH and 294 from NTUH) were enrolled in total and were surveyed for nasal carriage of MRSA after giving their written informed consent.

Detailed demographic data are shown in Table 1. Five hundred thirty-one were male (96%) and 22 female (4%). The majority of the participants were aged 20–60 years (with 47.6% between 20 and 39 years, and 45.6% between 40 and 60 years). The main route of HIV acquisition was male-to-male sex contact (68.0%), followed by injection drug use (16.3%). Table 1 shows the comparison among different hospitals and no statistically significant difference was found among different hospitals in terms of nasal MRSA carriage rate among the HIV-infected patients.

**Table 1:** Comparison of demographics and transmission vehicles between HIV-infected patients with and without methicillin-resistant *Staphylococcus aureus* (MRSA) nasal colonization

Characteristic	No. (%) of subjects			*P* value
	Total (*n* = 553)	MRSA carriers (*n* = 19)	Non-MRSA carriers (*n* = 534)	
Age in years, mean (SD)	41.2 (11.4)	43.8 (13.0)	41.1 (11.3)	0.296
Gender				
Male	531 (96)	15 (79)	516 (97)	0.005^*^
Female	22 (4)	4 (21)	18 (3)	
Route of HIV transmission				
Injection drug use	90 (16)	9 (47)	81 (15)	0.001^*^
Heterosexual activity	62 (11)	2 (11)	61 (11)	1.000
Male-to-male sex	375 (68)	6 (32)	369 (69)	0.002^*^
Blood transfusion	3 (0.5)	0 (0)	3 (0.6)	1.000
Vertical transmission	1 (0.2)	0 (0)	1 (0.2)	1.000
Unknown	36 (6.5)	2 (11)	34 (6.4)	0.380
Facility				0.203
CGMH, Linko branch	111 (20.1)	3 (15.8)	108 (20.2)	
CGMH, Taipei branch	13 (2.4)	0 (0)	13 (2.4)	
CGMH, Keelung branch	117 (21.2)	8 (42.1)	109 (20.4)	
Keelung jail and prison	18 (3.3)	1 (5.3)	17 (3.2)	
NTUH	294 (53.2)	7 (36.8)	287 (53.8)	

*Abbreviations*: *CGMH* Chang Gung Memorial Hospital, *NTUH* National Taiwan University Hospital

*significant difference (*p* < 0.05)

The overall MSSA and MRSA nasal colonization rate was 21.5 and 3.4%, respectively. The comparison of underlying diseases and other medical history between patients with and without MRSA colonization are shown in Tables 2 and 3. In univariate analysis, we found that female gender (*p* = 0.005), injection drug use (*p* = 0.001), smoking (*p* = 0.02), hepatitis C virus (HCV) carrier (*p* = 0.003), cancer (p = 0.001) and antibiotic use within 1 year (*p* = 0.02) were significant factors associated with MRSA colonization. CD4 + T lymphocytes or plasma HIV RNA load was not significantly associated with MRSA colonization. By multivariate analysis, cancer (adjust odds ratio (aOR) 7.78, [95% confidence interval (CI), 1.909–31.731]) and antibiotic use within 1 year (aOR 3.89, [95% CI, 1.219–12.433]) were significantly associated with MRSA colonization.

**Table 2:** Analysis of risk factors for nasal colonization with methicillin-resistant *Staphylococcus aureus* (MRSA) among HIV-infected outpatients

Characteristic	No. (%) of subjects			*P* value
	Total (*n* = 553)	MRSA carriers (*n* = 19)	Non-MRSA carriers (*n* = 534)	
**Personal history**				
Smoking	290 (52)	15 (79)	275 (51)	0.020^*^
Alcohol Drinking	139 (25)	6 (32)	133 (25)	0.590
Hospitalization after HIV diagnosis	269 (49)	7 (37)	262 (49)	0.354
Recent hospitalization within 1 year	80 (14)	5 (26)	75 (14)	0.174
Operation history	16 (3)	0 (0)	16 (3)	1.000
Nurse home	3 (0.6)	0 (0)	3 (0.6)	1.000
**Underlying diseases**				
Hypertension	59 (11)	3 (16)	56 (10)	0.443
DM	21 (4)	1 (5)	20 (4)	0.527
Arrhythmia history	10 (2)	1 (5)	9 (2)	0.297
Angina history	7 (1)	0 (0)	7 (1)	1.000
Pneumonia history	26 (5)	1 (5)	25 (5)	0.606
COPD	6 (1)	0 (0)	6 (1)	1.000
Asthma	12 (2)	0 (0)	12 (2)	1.000
Tuberculosis	20 (4)	1 (5)	19 (4)	0.509
HBV carrier	62 (11)	2 (11)	60 (11)	1.000
HCV carrier	123 (22)	10 (53)	113 (21)	0.003^*^
Cirrhosis	3 (0.5)	1 (5)	2 (0.4)	0.100
CKD	10 (2)	1 (5)	9 (2)	0.297
Cancer	27 (5)	5 (26)	22 (4)	0.001^*^
**Recent infectious diseases (within 3 months)**				
Recent URI	62 (11)	2 (11)	60 (11)	1.000
Skin infection	29 (5)	1 (5)	28 (5)	1.000
Unhealed wound	16 (3)	1 (5)	15 (3)	0.433
UTI	6 (1)	0 (0)	6 (1)	1.000
**Recent treatment (in 3 months)**				
On all catheters	10 (2)	1 (5)	9 (2)	0.297
Hemodialysis	2 (0.3)	1 (5)	1 (0.2)	0.068
Antibiotic use in 1 year	88 (16)	7 (37)	81 (15)	0.020^*^
**Laboratory data**				
CD4 count (cells/uL), mean ± SD		469.16 ± 214.8	540.61 + 292.3	0.292
Plasma HIV RNA load (Log_10_ of copies/ml), mean ± SD		1.341 ± 2.055	0.929 ± 1.660	0.293

*Abbreviations*: *DM* diabetes mellitus, *COPD* chronic obstructive pulmonary disease, *HBV* hepatitis B virus, *HCV* hepatitis C virus, *CKD* chronic kidney disease, *URI* upper respiratory infection, *SD* standard deviation

*significant difference (*p* < 0.05)

**Table 3:** Multivariate analysis of factors associated with nasal colonization of MRSA in HIV carriers in Taiwan

Factor	Adjust odds ratio	95% confidence interval	*P* value
Female Gender	2.910	0.690–12.276	0.1459
Injection drug user	1.189	0.207–6.837	0.8464
Male-to-male sex	0.241	0.055–1.050	0.0581
Smoking	2.004	0.581–6.911	0.2713
HCV carrier	2.233	0.504–9.885	0.2899
Cancer	7.782	1.909–31.731	0.0042
Antibiotic use within the past 1 year	3.892	1.219–12.433	0.0218

*Abbreviations*: *HCV* hepatitis C virus

**Fig. 1:**
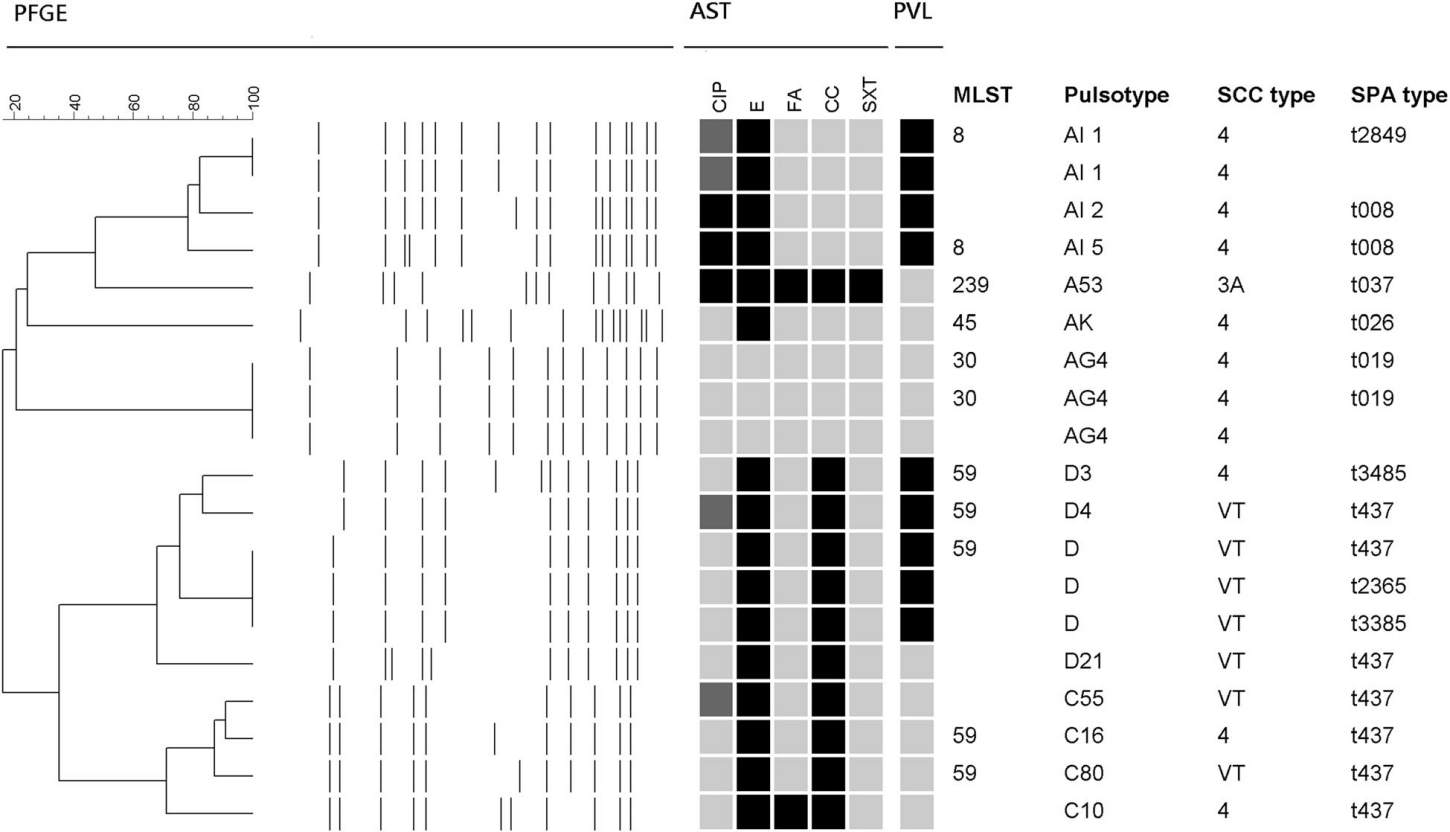
Molecular characteristics of methicillin-resistant *Staphylococcus aureus* isolates from 19 HIV-infected patients. All 19 isolates were resistant to penicillin, and susceptible to vancomycin, teicoplanin, linezolid, and doxycycline. Antimicrobial susceptibility tests (AST): black indicates resistance, and grey indicates susceptibility. Abbreviations are as follows: ciprofloxacin (CIP), erythromycin (E), fusidic acid (FA), clindamycin (CC), trimethoprim-sulfamethoxazole (SXT). PFGE, pulsed-field gel electrophoresis. PVL: black indicates that Pantone-Valentine leucocidin genes were detected. SCC*mec*, staphylococcal cassette chromosome *mec*; MLST, multilocus sequence typing. *Spa* types t2365, t3385 and t3485 are variants of t437, while t2849 is the variant of t008

All 19 MRSA isolates were available for molecular characterization and the distribution of PFGE patterns, SCC*mec* types, SPA typing, and the presence of PVL genes among the 19 MRSA isolates are shown in Fig. 1. MLST was selectively done in 11 isolates, and five sequence types were identified. Totally six PFGE patterns were identified. One isolate carried SCC*mec* type IIIA and the remaining isolates carried either SCC*mec* type IV or VT. PFGE pattern D/ sequence type (ST) 59 was the relatively common clone, followed by PFGE pattern C/ST 59 and PFGE pattern AI/ST 8. PVL is one of the most important virulence factors of *S. aureus* and PVL genes were present in 9 isolates (47%). PVL genes were present in all isolates with PFGE pattern AI/ST 8 and most isolates with PFGE pattern D/ST 59/SCC*mec* V_T_, but absent in isolates with PFGE pattern AG4/ST 30 and C/ST 59/SCC*mec* IV.

All the MRSA strains were resistant to penicillin and susceptible to linezolid, teicoplanin, doxycycline, and vancomycin. The susceptibility rates to erythromycin, clindamycin, ciprofloxacin, trimethoprim-sulfamethoxazole (TMP-SMX), and fusidic acid were 15.8, 42.1, 84.20, 94.7, and 89.5%, respectively.

## Discussion

To our knowledge, this is the largest study on this issue in Asian countries [15]. In this cross-section survey among 553 HIV-infected patients seeking care at the outpatient clinics in four hospitals in northern Taiwan, we found that the overall prevalence of MRSA nasal carriage among the HIV-infected individual was 3.4% while the prevalence of MSSA nasal carriage was 21.5%. The nasal carriage of MRSA was associated with cancer and antibiotic exposure within the past 12 months.

The nasal carriage rate of MRSA in this study is slightly lower than that previously reported in Taiwan in 2003 (5.6% of 296 HIV-infected patients) [12] and in 2009–2010 (4.4% of 457 HIV-infected patients) [23]. Compared with other populations in Taiwan, the nasal MRSA carriage rate among HIV-infected patients was significantly lower than that for patients hospitalized in the intensive care units (ICU) (32% of 177 patients) [30], but similar to that for adult patients receiving hemodialysis (3.8% of 296 patients) [31], adult patients visiting emergency room (3.8% of 502 patients) [32] and otherwise healthy adults for health examination (3.8% of 3098 adults) [33]. It could be explained that in the era of highly active antiretroviral therapy (HAART), the majority of the HIV-infected patients recruited in this study came from community settings, outpatient visiting and these patients were not frequently exposed to the high-risk populations for MRSA acquisition, such as patients admitted to the ICUs.

For patients infected with HIV, the nasal MRSA carriage rate was different, with a huge discrepancy, in different countries and regions. A latest meta-analysis on this issue [15] showed that the estimated pooled worldwide prevalence of MRSA in HIV-infected people is around 7% (5–9%), with the prevalence of 7–13% in the region of the Americas and 0–1% in the European region. Compared with other Asian countries, the prevalence rate (3.4%) in this study was lower than that in India (6–26%), as well as in Singapore (3–10%) but higher than that in Malaysia (< 1%). This variation may be attributable to different prevailing MRSA clones, study population, the intermittent nature of colonization [10] and different culture of antibiotic use between countries [34].

We observed that MRSA colonization was associated with antibiotic use within 12 months among HIV-infected individuals. This finding supported previous observations that recent receipt of antibiotics was strongly associated with MRSA colonization [12]. Wang et al. found that smoking was a protective factor against MRSA colonization in the community setting [33], which was not confirmed in this study. The potential reasons for the disparity might be the difference in questionnaire design. A latest meta-analysis on this issue [15] indicated that the risk factors for MRSA colonization included having a previous MRSA infection, hospitalization in the past year and use of antibiotics in the past 6 months.

Molecular characterization of MRSA isolates in the present study showed that 10 (52.6%) of the 19 isolates belonged to ST59 linage, the predominant community-associated MRSA strain in Taiwan. One isolate was characterized by ST239-SCC*mec* IIIA which had been known as the most dominant healthcare-associated MRSA clone in Taiwan [4]. The patient with the isolate of ST239 did have a medical history of recent hospitalization within the past year and had been admitted four times after being diagnosed with HIV. The remaining eight isolates carried type IV SCC*mec*. Among these eight isolates, four of them were identified and confirmed as USA 300 later [35], the predominant community-associated MRSA strain in the United States, on the basis of their being sequence type 8 by multilocus sequence typing (MLST), detection of arginine catabolic metabolic element (ACME) gene and harboring SCC*mec* type IV and genes for PVL. Three of them were characterized by ST30-SCC*mec* IV strains, known as the Southwest Pacific clone [2, 4]. One isolate characterized as sequence type 45 which had been identified as the predominate strain in nursing homes in Taiwan [36]. In this study, USA300 (ST8) accounted for four isolates and was a second most common clone. All four isolates were from NTUH. This is an emergent issue in Taiwan [35], which needs further surveillance and observation.

There were several limitations to the current study. First, less than 70% of the HIV-infected patients visiting CGMH and NTUH participated in this study which reduced the sample size and indirectly affected the analysis of statistical significance. It may be the reason for the lack of statistically significant association between MRSA colonization and the common risk factors reported in previous studies such as low CD4 T-cell count and hospitalization within the past year [37]. Second, the presence of other unrecognized sites of colonization should be considered. In this study, nasal specimens for MRSA detection were obtained by swabbing both anterior nares. Patients might be colonized in the inguinal, genital or peri-rectal areas but not in the nasopharynx [11, 37]. Other potential reasons for the disparity between the studies included the intermittent nature of colonization [10] and the difference in questionnaire design.

## Conclusions

The prevalence of nasal MRSA colonization in HIV-infected patients seeking outpatient care was low (3.4%) in northern Taiwan. Most of the colonizing isolates were genetically endemic community strains and exhibited high susceptible to TMP-SMX and fluoroqinolones. Cancer and antibiotic use within 1 year were associated with MRSA colonization. Nasal MRSA carriage in HIV-infected patients seeking outpatient care was low (3.4%) in northern Taiwan.

## Data Availability

The datasets used and/or analysed during the current study are available from the corresponding author on reasonable request.

## Abbreviations

MRSA: Methicillin-resistant *Staphylococcus aureus*
MSSA: Methicillin-sensitive *Staphylococcus aureus*
CA: Community-associated
PFGE: Pulsed-field gel electrophoresis
MLST: Multilocus sequence typing
ST: Sequence type
PVL: Panton-Valentine leucocidin
ACME: Arginine catabolic metabolic element
HIV: Human immunodeficiency virus
HCV: Hepatitis C virus
ICU: Intensive care units
